# Gender Differences in the Relationship between Living alone and Depressive Symptoms in Elderly Korean Adults

**Published:** 2019-03

**Authors:** Young Bum KIM, Seung Hee LEE

**Affiliations:** 1. Institute of Aging, Hallym University, Hallymdaehak-gil, Chuncheon, Gangwon-do 200-702, Republic of Korea; 2. Department of Nursing, University of Ulsan, Nam-gu, Daehak-ro 93, Ulsan 680-749, Republic of Korea

**Keywords:** Living arrangement, Depressive symptom, Social activity, Support, Elderly, Korea

## Abstract

**Background:**

This study examined gender differences in the relationship between living alone and depressive symptoms in elderly Korean adults and the variables that influence this relationship.

**Methods:**

We conducted a secondary analysis using fourth-wave data from adults 65 yr of age or older who participated in the Korean Longitudinal Study of Aging. Depressive symptoms were measured using the 10-item short-form of the Center for Epidemiological Studies Depression Scale. Multiple logistic regression analyses were used.

**Results:**

After controlling for the factors of formal and informal social activities, financial support from children, employment, activities of daily living, instrumental activities of daily living, self-rated health, frequency of meals, and gender, living alone (AOR=1.45, 95% CI =1.09–1.93, *P*=0.010) was an independent risk factor that contributed to depressive symptoms in late life. Living alone was more likely to elevate depressive symptoms in older women but not in older men.

**Conclusion:**

Gender differences in the depressive effect of living alone in late life may differ across diverse cultures.

## Introduction

South Korea is one of the most rapidly aging countries in the world. In 2015, the number of people aged 65 or older in the country reached 6.6 million, accounting for 13.1% of the total population, and it is projected to increase to 30% by 2037 (1). It was also reported in 2015 that 20.6% of older persons aged 65 or older lived alone (1). As a result of family structural changes, urbanization, and weakened filial piety, the number of elderly living alone is steadily increasing in developed Asian countries such as South Korea, Japan, and China (2–4).

Living alone has been found to be positively associated with specific health concerns, such as poorer health habits (5), lower levels of psychological well-being (6), institutionalization (7), and mortality (7), among older adults. Elderly people who reside alone are more likely to be female, widowed, and unemployed and have poorer self-rated health and more problems with physical function (3, 8). Elderly people living alone were more likely to report a higher level of depressive symptoms than those who did not (4, 6, 8). Scholars have also noted the importance of considering gender and marital status when distinguishing the influence of living alone on depression (9). Recent studies on gender differences in this topic have been somewhat inconsistent in their findings. For example, depressive symptoms were more common in elderly men living alone than in elderly females (10, 11). In contrast, elderly women who live alone are at an increased risk for depressive symptoms after their husband’s death (8, 12, 13). A few studies have also identified no significant gender differences in the association between living alone and depressive symptoms in the elderly (6). Existing studies on gender differences in elderly people who reside alone have reported that living alone in men is associated with poorer diet, poorer self-rated health, and less help to children (10, 14), while in women, living alone is associated with greater financial strain, physical limitations, and more help from children (8, 10). Moreover, elderly women living alone are neither socially isolated nor at high risk for a decline in their functional health status (15, 16). Instead, elderly women who reside on their own report more contact with close friends outside the household (15). These gender differences regarding characteristics of living alone may have relevance to gender differences in the relationship between living alone and depressive symptoms in the elderly. However, in previous studies on the relationship between living alone and depressive symptoms among older men and women, the aforementioned variables (e.g., diet, social activities and financial support from children) have rarely been considered as confounding factors (8, 17). Therefore, because considerable inconsistencies exist in the findings from the previous literature, more research is needed to explore gender differences in the association between living alone and depressive symptoms and also to better understand related factors that underlie this relationship. Nevertheless, little is known about gender differences with regard to this topic among older adults in South Korea.

Therefore, the aim of this study was to examine the gender differences that affect the relationship between living alone and depressive symptoms, including various important potential confounders in the analysis, using a large and nationally representative sample of older Korean adults.

## Methods

### Study design and participants

The current study was a secondary analysis that used fourth-wave data from the Korean Longitudinal Study of Aging (KLoSA), an ongoing panel survey examining the socioeconomic, physical, and psychological aspects of aging among community-dwelling Koreans 45 yr of age or older. The sample was stratified based on age and gender. To ensure national representation, participants were randomly selected using a multistage, stratified probability sampling design from household units chosen according to geographical area, including both urban and rural areas. Before the data collection, all participants provided written informed consent.

In the 2012 KLoSA, 7486 individuals completed interviews conducted by a skilled interviewer using a structured questionnaire. The baseline survey response rate was 80.1%. Of the data from the baseline survey, participants who were younger than 65 yr of age (n=3352); never married, divorced, or separated (n=47); without children (n=76); living only with children (n=107), or had missing data on depression or income (n=110) were excluded. Data from the remaining 3794 respondents who were 65 yr of age and older were used in this analysis. The mean age of the sample was 74.5 yr (SD =6.5, range=65–103). Of the 3794 participants included in the current study, 5.1% of men and 25.1% of women lived alone, for a total of 16.6%. Weights were assigned to reduce the sampling error introduced by stratified sampling. More sampling design details and recruitment procedures of the KLoSA have been presented elsewhere (18, 19).

### Measures

#### Living arrangements

The living arrangements of respondents were categorized into two groups according to the study questionnaire: living alone and not living alone. The participants were asked whether they resided alone or with others.

#### Depressive symptoms

The 10-item short-form of the Center for Epidemiological Studies Depression (CES-D10) Scale was used to measure the level of depressive symptoms in the respondents. This inventory is widely used to detect depressive symptoms in non-psychiatric community settings (19, 20). The CES-D10 assesses self-reported depressive symptoms experienced during the two weeks prior to testing. Total scores ranged from 0–30. Higher scores indicate more symptoms of depression. In previous CES-D10 validation studies, the cutoff value between moderately severe and severe depression was identified as 10 points (20). Thus, this study used a standard cutoff score of 10 to categorize individuals as depressed. Psychometric properties of the CES-D10 have been validated in previous studies (20, 21). The internal consistency of the CES-D10 in the current sample was good (Cronbach’s α = 0.87).

#### Covariates

Sociodemographic variables included age, gender, marital status, education, monthly household income, financial support from children, employment status, and residential area. Monthly household income was calculated as the total household income divided by the square root of the number of household members. Financial support from children was measured by the question “Do you receive any financial help from your children?” Responses were coded as either “yes” or “no.”

Health-related variables included comorbidities (hypertension, diabetes mellitus, heart disease, stroke, and cancer), activities of daily living (ADL), instrumental activities of daily living (IADL), self-rated health, frequency of meals, and cognitive function. Cognitive function was evaluated using the Mini-Mental State Examination (MMSE). Total scores ranged from 0–30. Lower scores indicated a greater degree of cognitive impairment.

Social activities were divided into formal and informal based on intimacy and the intensity of participation required (22). We employed four variables to represent levels of participation in the following formal social activities: (a) church or other religious groups, (b) senior citizen clubs or senior centers, (c) alumni societies or family councils, and (d) sports, art, or music groups. Participants rated how often they took part in each activity based on a 10-point scale (0=never; 1=almost never; 2=less than once a year; 3=1–2 times a year; 4=3–4 times a year; 5=5–6 times per year; 6=once a month; 7=twice a month; 8=once a week; 9=2–3 times a week; and 10=every day or almost every day). We employed three variables to represent informal social activities: (a) level of face-to-face contact with close friends, (b) level of face-to-face contact with children, and (c) level of contact with children by phone or letter (19). Participants answered the following three questions: 1) How often do you meet with your close friends? 2) How often do you meet with your adult children? And 3) How often do you contact your children by phone or letter? These were assessed using the same 0–10-point scale.

### Statistical analysis

Characteristics of the respondents were summarized as the frequency, percentage, and mean by living arrangement. Adjusted multiple logistic regression analyses were conducted to evaluate any differences in the relationships between living arrangements and depressive symptoms after controlling for socio-demographic and health-related variables and social activities. Multicollinearity was checked for all study variables using correlations and collinearity statistics tests (tolerance and variance inflation factor tests). The correlations were sufficiently low (r<0.71), and all VIF scores were below 4, allowing for entry of all variables into multiple logistic regression models (23). Statistical analyses were conducted using the Stata software (version 14). All analyses were reported as two-tailed with α set at 0.05.

## Results

Table 1 presents the effects of living alone on all independent variables and depression symptom levels in the total sample.

**Table 1:** Characteristics of the sample by living arrangement

***Variables***	***Not Living alone (n=3156)***	***Living alone (n=638)***	***t or X^2^***	***P-value***
	***Mean ± SD or n (%)***	***Mean ± SD or n (%)***		
**Socio-demographics**				
Age (yr)	74.1±6.6	76.3±6.1	−7.75	< .001
Gender			271.19	< .001
Female	1,625(51.5)	554(86.8)		
Male	1,531(48.5)	84(13.2)		
Marital status			1.600	< .001
Bereaved	579(18.4)	638(100.0)		
Married	2,577(81.6)	0(0.0)		
Education			99.74	< .001
≤Elementary school	1,959(62.1)	527(82.6)		
Middle school	450(14.3)	47(7.4)		
High school	522(16.5)	47(7.4)		
College or above	225(7.1)	17(2.7)		
Monthly household income (×1,000 Korean won^†^)	1,313.4±1,459.1	752.9±1,642.4	8.66	< .001
Financial support from Children			2.00	.158
No	352(11.2)	59(9.3)		
Yes	2,804(88.8)	579(90.7)		
Employment			18.91	< .001
No	2,380(75.4)	532(83.4)		
Yes	776(24.6)	106(16.6)		
Residential area			7.28	.007
Rural	966(30.6)	230(36.1)		
Urban	2,190(69.4)	4.8(63.9)		
**Health related variables**				
Comorbidities				
Hypertension			11.05	.001
No	1,593(50.5)	276(43.3)		
Yes	1,563(49.5)	362(56.7)		
Diabetes mellitus			0.32	.574
No	2,490(78.9)	497(77.9)		
Yes	666(21.1)	141(22.1)		
Cancer			0.39	.531
No	2,999(95.0)	610(95.6)		
Yes	157(5.0)	28(4.4)		
Heart disease			0.03	.855
No	2,793(88.5)	563(88.2)		
Yes	363(11.5)	75(11.8)		
Stroke			4.15	.042
No	2,904(92.0)	602(94.4)		
Yes	252(8.0)	36(5.6)		
Activity of daily living	0.4±1.4	0.2±1.1	2.49	.013
Instrumental Activity of daily Living	1.0±2.5	0.6±2.0	3.50	< .001
Self-rated health	3.3±0.9	3.5±0.8	−3.62	< .001
Frequency of meal for the last two days			24.14	< .001
Less than 6 times	155(4.9)	63(9.9)		
Six times	3,001(95.1)	575(90.1)		
MMSE score	21.9±7.8	20.6±7.2	3.82	< .001
**Social activities**				
Formal social activity				
Level of participation in church or other religious groups	1.6±3.2	1.7±3.3	−0.81	.418
Level of participation in senior citizens’ club or senior center	3.5±3.7	3.4±4.1	0.91	.365
Level of participation in alumni society or family councils	.6±1.7	.2±1.0	5.46	< .001
Level of participation in sports, art or music groups	.3±1.5	.3±1.7	−1.12	.263
Informal social activity				
Level of face to face contact with close friends	7.5±2.7	8.1±2.5	−5.36	< .001
Level of face to face contact with children	5.1±1.4	5.1±1.5	0.32	.745
Level of contact by phone or letter with children	7.1±1.4	7.1±1.4	0.35	.727
**Depressive symptoms**				
CES-D score	8.2±5.7	9.6±5.9	−5.71	< .001
Percent of depression (CSE-D≥10)	1,133(35.9)	308(48.3)	34.51	< .001

Note: MMSE = Mini-Mental State Examination, CES-D = Center for Epidemiological Studies Depression,

†: The 1,000 Korean Won corresponds to 1 US dollar

Elderly adults who lived alone were likely to be more depressed, older, female, and have lower household incomes, but they were less likely to report regularly eating three meals a day compared to those who lived with family members.

In our sample, all older adults who lived alone were bereaved. Table 2 shows the results of the multiple logistic regression analysis with living arrangement as the independent variable and depressive symptoms as the dependent variable. Older adults living alone had significantly greater odds ratios (ORs) of depressive symptoms compared to those who resided with family members.

**Table 2:** Multiple logistic regression analysis of the association between living alone and depressive symptoms in Korean elderly: total sample

***Variables***	***AOR(95%CI)^a^***	***P-value***
**Socio-demographics**		
Age (yr)	0.99(.98 1.01)	.297
Marital status(Bereaved=0)		
Married	0.83(.64 1.09)	.187
Live alone(0=not live alone)		
Live alone	1.45(1.09 1.93)	.010
Gender(female=0)		
Male	0.94(.77 1.16)	.569
Education(≤Elementary school=0)		
Middle school	1.04(.78 1.36)	.756
High school	1.02(.78 1.32)	.909
College or above	1.30(.87 1.96)	.205
Monthly household income (×1,000 Korean won^†^)	1.00(.99 1.00)	.594
Financial support from children(0=no)		
yes	1.43(1.09 1.88)	.010
Employment (no=0)		
yes	0.59(0.48 0.74)	< .001
Residential area(Rural=0)		
Urban	1.02(0.84 1.23)	.829
**Health related variables**		
Hypertension(no=0)		
Yes	1.05(0.89 1.24)	.573
Diabetes mellitus(no=0)		
Yes	0.98(0.86 1.20)	.866
Cancer(no=0)		
Yes	1.07(0.75 1.52)	.707
Heart disease(no=0)		
Yes	1.00(0.78 1.30)	.973
Stroke(no=0)		
Yes	1.15(0.84 1.59)	.379
Activity of daily living	0.91(0.81 1.03)	.145
Instrumental Activity of daily Living	1.09(1.02 1.17)	.011
Self-rated health(1=very good, 5=very bad)	1.73(1.54 1.94)	< .001
Frequency of meal for the last two days (less than 6 times=0)		
6 times	0.52(0.36 0.77)	.001
MMSE score	0.96(0.95 0. 97)	< .001
**Social activities**		
Formal social activity		
Level of participation in church or other religious groups	1.01(0.99 1.04)	.416
Level of participation in senior citizens’ club or senior center	1.01(0.99 1.03)	.446
Level of participation in alumni society or family councils	0.98(0.93 1.04)	.565
Level of participation in sports, art or music groups	0.94(0.89 1.10)	.047
Informal social activity		
Level of face to face contact with close friends	0.91(0.88 0.94)	< .001
Level of face to face contact with children	1.09(1.01 1.68)	.022
Level of contact by phone or letter with children	0.76(0.71 0.82)	< .001

Gender was not a significant predictor of depressive symptoms. Being dependent on others for IADLs, having poor self-rated health, receiving financial support from children, and having frequent face-to-face contact with children were all positively associated with depressive symptoms in older adults. Being employed, having higher MMSE scores, eating six meals during the last two days, participating in sports, art, or music groups, having face-to-face contact with close friends, and contacting children by phone or letter were negatively associated with depressive symptoms in older adults.

Living alone was a risk factor for the presence of depressive symptoms in women but not in men. Self-rated health, financial support from children, and face-to-face contact with children were significant risk factors for depressive symptoms in women. Elderly women with poor self-rated health who received financial support from their children and regularly engaged in face-to-face contact with their children were more likely to experience depressive symptoms than their counterparts. In men, poor self-rated health and being dependent in IADLs were significant risk factors for depressive symptoms.

## Discussion

Our study revealed that living alone had a statistically reliable effect on depressive symptoms independently of any effects of formal and informal social activities, financial support from children, employment, ADLs, IADLs, self-rated health, frequency of meals, and gender, which was consistent with the results of previous studies (6, 8). This finding may also be explained by the Asian cultural context and preference of elderly individuals for living with their adult children. In Asian culture, filial piety is highly valued, and adult children are expected to take care of their elderly parents (8, 12). Family is an important source of support for older adults in the Asian culture (12). Cohabiting with family can help older adults be socially connected, and social support from family plays an important role in maintaining life satisfaction in older adulthood (24, 25). Thus, it is preferred for older adults to live with their children rather than to live alone after their spouse’s death (8). However, in recent decades, industrialization and urbanization have resulted in changes in family structure, so many adult children no longer live with their parents in South Korea (19). These changes have eroded older adults’ expectation of residing with their adult children and failed to meet their desire for family closeness and support, which may result in feelings of insecurity and loneliness while also producing depressive symptoms (13). Compared with those who reside with family, elderly people who live alone tend to have fewer chances to feel intimate emotions and belonging because they have less contact with and instrumental support from their family (4, 25). This decrease may be one reason why elderly people who live alone demonstrate a higher level of depressive symptoms than those who live with family.

In our study, an impact of living alone on depressive symptoms was only found in older women but not for older men. This finding is inconsistent with the results of studies in Western countries, reported that living alone has a more adverse effect on men than on women (10, 14). In Western countries, living alone elevates depressive symptoms more for men than for women because marriage is a stronger barrier against depressive symptoms for men (10, 11). Nevertheless, several studies in China and Taiwan have shown that the impact of living alone on depressive symptoms is evident in older women but not in older men (8, 13), which is consistent with our findings. One possible explanation for this difference may be that in Asian culture, the emotional bond between parents and children is more essential for women than it is for men. Females have stronger emotional connections with and are more dependent upon their children than are males in Asian society (12, 25). After bereavement, older women may desire a more intimate relationship with their children as an alternative to a male spouse, and living together is one way to achieve this more intimate relationship (4, 8). Therefore, the negative effect of living alone may be stronger for older women than it is for older men. Our findings in older women may be a result of emotional ties and dependence that females have with their children. Another possible explanation for our finding may be a Type II error—a failure to detect a relationship between living alone and depressive symptoms in men because the sample size of elderly men who lived alone (n= 84) was too small.

After the death of a spouse, older women experience greater financial difficulties than older men, and this financial strain may increase the likelihood of depressive symptoms among these women (8, 10). In the present study, financial support from children was an independent risk factor that contributed to depressive symptoms in elderly women. This result may be due to their perceived financial vulnerability and also the desire not to burden their grown children.

Our findings revealed that frequent face-to-face contact with their children was positively associated with depressive symptoms among elderly women. In Korea, the high female employment rate has resulted in an increased need for assistance with household affairs, such as taking care of young children in the family (19). When requested, most elderly women will meet with and provide instrumental support for their grown children (12, 18). The burdens of helping their adult children could lead to chronic stress and depressive symptoms in older women (19).

Formal and informal social activities are considered to be significant protective factors against depressive symptoms later in life (12). Through social interaction with close friends, older adults may feel less lonely and be more likely to receive emotional support, an essential shield against depression (9).” Talking with their adult children on the phone is likely to provide older adults with perceived emotional support and intimacy “(26). The present study had several limitations. First, because of the small number of elderly men who lived alone, we did not identify any association between living alone and depressive symptoms in older men. Future studies should include a large enough sample size to provide a more robust idea of the effects of living alone on depressive symptoms in older men. Second, this study did not include information on how long the older adults had been in their current living arrangement, even though depressive symptoms in the elderly may be affected differently by the duration of their present living situation (10). Third, we relied on self-reported survey data, which are subject to errors of recall or the tendency to provide socially desirable responses (27). Lastly, our study was based on cross-sectional data, so we cannot rule out any causal conclusions. Further longitudinal studies are needed to explore the causal and temporal relationship of living alone on depressive symptoms.

Despite these limitations, this study provided evidence that living alone is an independent risk factor that contributes to depressive symptoms, in a large, nationally representative sample of elderly Koreans.

## Conclusion

After adjusting for various potential confounders, this community-based study indicated that living alone is an independent risk factor that contributes to depressive symptoms, and living alone is more likely to produce depressive symptoms in older women but not in older men. Gender differences in the depressive effect of living alone later in life may vary across diverse cultures, especially in Asian societies. Sufficient formal and informal social activities, good health, cognitive function, and work activities could be protective factors against developing depressive symptoms in older adults, regardless of their living arrangements.

## Ethical considerations

Ethical issues (Including plagiarism, informed consent, misconduct, data fabrication and/or falsification, double publication and/or submission, redundancy, etc.) have been completely observed by the authors.
